# A Case of Descending Necrotizing Mediastinitis in a Previously Healthy Child

**DOI:** 10.1155/2021/3159092

**Published:** 2021-09-16

**Authors:** Toshihiko Okumura, Nobuyuki Tetsuka, Makoto Yamaguchi, Takako Suzuki, Yuka Torii, Jun-ichi Kawada, Yoshinori Ito

**Affiliations:** ^1^Department of Pediatrics, Nagoya University Graduate School of Medicine, 65 Tsurumai-cho, Showa-ku, Nagoya 466-8550, Japan; ^2^Department of Infectious Disease, Nagoya University Hospital, 65 Tsurumai-cho, Showa-ku, Nagoya 466-8550, Japan

## Abstract

Descending necrotizing mediastinitis (DNM) is a rare complication of oropharyngeal and cervical infection, especially in children. We report a case of DNM secondary to a cervical abscess in a previously healthy 1-year-old boy. The patient presented with redness and swelling of the neck and fever. He was treated with an antimicrobial agent for the diagnosis of cervical lymphadenitis. On the sixth day, a huge mediastinal abscess was found, and he was admitted to the intensive care unit. He was successfully treated with surgical drainage and appropriate antimicrobial therapy. The pus culture isolated multiple bacteria, including methicillin-resistant *Staphylococcus aureus* (MRSA). Although we did not use an antimicrobial agent covering MRSA, the symptoms and test results improved. Washing with drainage was effective. The patient required multidisciplinary treatment, and we collaborated with specialists in other departments. DNM is a severe disease in which team medical care is needed to provide appropriate treatment.

## 1. Introduction

Descending necrotizing mediastinitis (DNM) is a rare complication of oropharyngeal and cervical infection, especially in children. We report a case of DNM secondary to a cervical abscess in a previously healthy 1-year-old boy. The patient presented with redness and swelling of the neck and fever. He was treated with an antimicrobial agent for the diagnosis of cervical lymphadenitis. On the sixth day, a huge mediastinal abscess was found, and he was admitted to the intensive care unit. He was successfully treated with surgical drainage and appropriate antimicrobial therapy. The pus culture isolated multiple bacteria, including methicillin-resistant *Staphylococcus aureus* (MRSA). Although we did not use an antimicrobial agent covering MRSA, the symptoms and test results improved. Washing with drainage was effective. The patient required multidisciplinary treatment, and we collaborated with specialists in other departments. DNM is a severe disease in which team medical care is needed to provide appropriate treatment.

## 2. Case Presentation

Descending necrotizing mediastinitis (DNM) is a severe acute inflammation of the mediastinum secondary to oropharyngeal and cervical infection spreading rapidly through the space between cervical fasciae. Common primary origins include tonsillar and pharyngeal infection and odontogenic infection [1, 2]. DNM is a rare complication and has a high mortality rate of 6–15% as reported in recent studies [1–3]. Although the mortality rate in children may be lower than that in adults [4–7], DNM is a clinically important disease that requires early diagnosis and appropriate treatment because it can cause septic shock and airway emergency. We report the case of a child with DNM who was successfully treated without permanent damage despite the presence of a huge mediastinal abscess. Written informed consent was obtained from the patient's guardians for publication of this case report and the accompanying images.

**Figure 1:**
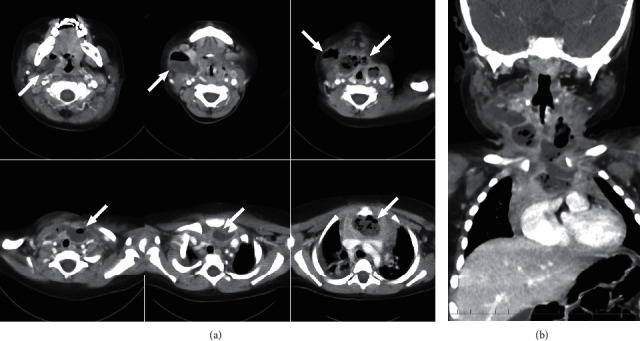
Axial (a) and coronal (b) images from the contrast-enhanced CT scan of the head and chest on admission. The white arrows show an abscess ranging from the neck to the anterior mediastinum.

A 1-year-and-5-month-old previously healthy boy presented with redness and swelling of the neck and fever. He was admitted to a general hospital for the diagnosis of cervical lymphadenitis. Although ceftriaxone was administered at 60 mg/kg per day, the fever persisted, the right side of the neck became red, and swelling of the region worsened. On the sixth day, a contrast-enhanced computed tomography (CT) scan of the head revealed a right cervical abscess spreading to the mediastinum. He was admitted to our hospital for intensive care, including surgical management. On admission, his vital signs were as follows: temperature, 39.1°C; pulse rate, 112 beats/min; blood pressure, 77/42 mmHg; respiration rate, 34 times/min; and 90% oxygen saturation in ambient air. The results of the laboratory examinations were as follows: white blood cell count, 22,300/*μ*L; aspartate aminotransferase, 80 U/L; alanine aminotransferase, 60 U/L; C-reactive protein, 24.72 mg/dL; and procalcitonin, 2.1 ng/mL. A contrast-enhanced CT scan of the head and chest revealed an abscess in the right submandibular region and a sequential abscess of the anterior mediastinum (Figure 1) that touched the posterior surface of the sternum. The patient was therefore diagnosed with DNM associated with a cervical abscess.

**Figure 2:**
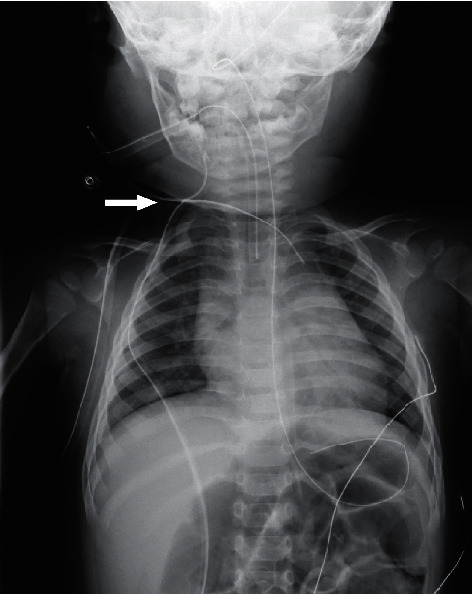
Chest radiograph after percutaneous drainage tubes were placed. The white arrow shows the insertion site. One was placed in the right neck, and the other was placed in the anterior mediastinum.

After admission to the intensive care unit, anesthesiologists anesthetized him and performed intratracheal intubation considering the possibility of tracheal obstruction. Pediatric surgeons performed percutaneous drainage from the neck and placed two drainage tubes (Figure 2). Foul-smelling cream-colored pus was drained. Pediatricians began intravenous administration of piperacillin/tazobactam (PIPC/TAZ) at 330 mg/kg per day for the purpose of broadening the antibacterial spectrum to include *Pseudomonas aeruginosa* and obligate anaerobes. On hospital day 2, PIPC/TAZ was changed to ampicillin/sulbactam at 300 mg/kg per day because the results of the pus culture ruled out *Pseudomonas aeruginosa*. On hospital day 4, several bacteria were identified, including a large amount of *Prevotella oris* and *Fusobacterium nucleatum* and a small amount of *Streptococcus constellatus*, *Eikenella corrodens*, and methicillin-resistant *Staphylococcus aureus* (MRSA). We did not add an antimicrobial agent to target MRSA because the high fever dropped, the results of blood examinations improved, and the abscess was reduced in size on the CT scan. The blood culture for only aerobes was negative. On hospital day 6, as the fluid from the drainage tubes gradually decreased and the tubes seemed to be clogged; pediatric surgeons replaced the drainage tubes with thicker tubes and washed out the cavity. On hospital day 11, the patient was extubated after an otolaryngologist assessed the edema of the respiratory tract. On hospital days 18 and 19, the drainage tubes were removed one by one. Antibiotic therapy in our hospital was continued for a total of 23 days after confirming that the patient did not develop osteomyelitis as a complication on magnetic resonance imaging. He was discharged on hospital day 26 without any complications.

## 3. Discussion

DNM is caused by the downward spread of oropharyngeal and cervical infections. Pharyngeal infection is the most frequent causative infection for DNM in adults [1, 2], and DNM is a rare complication that develops in 9% of deep neck infections [8, 9]. Meanwhile, in children, retropharyngeal abscess is the most frequent cause of DNM. DNM is a critical illness that requires appropriate treatment because pharyngeal and mediastinal abscesses can cause airway emergencies [10, 11]. Airway management is the most important treatment for deep neck abscesses and mediastinal abscesses. In this case, as the abscess reached the anterior aspect of the trachea and the oxygen saturation decreased, we emergently conducted intratracheal intubation. When the patient was extubated, an otolaryngologist evaluated the condition of the larynx so that airway obstruction due to edema after extubation would not occur.

The main symptoms of DNM are fever, odynophagia, cervical swelling, cervical pain, and dyspnea [1]. Infrequent symptoms include thoracic pain and back pain. It is difficult to diagnose DNM only based on clinical symptoms and physical examination because these symptoms are not specific to DNM. If a patient has cervical and chest symptoms, we must consider the possibility of DNM developing. It is very important to perform a CT scan when we suspect DNM to make an early diagnosis.

Prior studies reported that the most commonly isolated pathogen in pus cultures in children was *S. aureus*, most of which were MRSA [4, 5, 7]. Over 50% of adult patients with DNM were reported to have a polymicrobial infection with aerobic and anaerobic organisms [1, 3]. In this case, the pus culture isolated two anaerobes, *P. oris* and *F. nucleatum*, in addition to MRSA. Although we did not use an antimicrobial agent covering MRSA when MRSA was discovered, the symptoms and the test results tended to improve. We would have added an antimicrobial agent if the patient had become ill. Since the number of anaerobes was large, these bacteria were identified as the main pathogen, and antibiotic therapy and washing with drainage were considered sufficiently effective. It was suggested that MRSA detected in the culture does not necessarily have to be covered.

Drainage is very important for the treatment of DNM, and surgical management was performed in all cases in several studies [1, 3, 5]. As surgical management is required two times on average [2, 5], it should be remembered that the initial drainage is not always sufficient. It is necessary to always consider the possibility of relapse of the abscess and evaluate treatment efficacy using imaging tests, such as contrast-enhanced CT, as appropriate. In this case, the amount of pus from drainage tubes was decreased due to clogging; therefore, we placed thicker tubes again 5 days after the first drainage, and the clinical course of the patient was subsequently good.

In conclusion, this case report confirms that early diagnosis by a CT scan and early treatment with drainage, which is often needed multiple times, are important for the medical care of DNM. MRSA detected in pus culture may not always be covered. We should continue to accumulate case reports to further improve the treatment of DNM in children.

## Data Availability

All data used to support the findings of this study are available upon request to the corresponding author.
